# Polymeric immunoglobulin receptor deficiency attenuates experimental atherosclerosis

**DOI:** 10.3389/fimmu.2026.1774396

**Published:** 2026-04-23

**Authors:** Isabel Cerro-Pardo, Belén Picatoste, Irene Raposo-Gutiérrez, Inmaculada Martos-Folgado, Cristina Márquez-Gálvez, Ana García-García, Lucía Ortega-Villanueva, Sonia M. Mur, Iñaki Robles-Vera, Joan Carles Escolà-Gil, Estefanía Núñez, David Sancho, Jes Lindholt, Jesús Vázquez, Luis Miguel Blanco-Colio, Almudena R. Ramiro, José Luis Martín-Ventura

**Affiliations:** 1 Instituto de Investigación Sanitaria-Fundación Jiménez-Díaz- Autónoma University of Madrid (IIS-FJD, UAM), Madrid, Spain; 2 B-cell Lab, Centro Nacional de Investigaciones Cardiovasculares Carlos III, Madrid, Spain; 3 Immunobiology Lab, Centro Nacional de Investigaciones Cardiovasculares Carlos III, Madrid, Spain; 4 Pathophysiology of Lipid-Related Disease Lab, Institut de Recerca (IIB) Sant Pau, Barcelona, Spain; 5 CIBER de Enfermedades Metabólicas (CIBERDEM), Madrid, Spain; 6 Cardiovascular Proteomics Laboratory, Centro Nacional de Investigaciones Cardiovasculares Carlos III, Madrid, Spain; 7 CIBER de Enfermedades Cardiovasculares (CIBERCV), Madrid, Spain; 8 Department of Cardiothoracic and Vascular Surgery, Odense University Hospital, Elite Research Centre of Individualized Medicine in Arterial Disease (CIMA), Clinical Institute, University of Southern Denmark, Odense, Denmark

**Keywords:** atherosclerosis, germinal center (GC) B cell, immunoglobulins, macrophage, polymeric immunoglobulin receptor

## Abstract

**Background:**

Polymeric immunoglobulin receptor (PIGR) is a transmembrane protein widely expressed in mucosal epithelial cells that is involved in the transcytosis of the polymeric immunoglobulins IgA and IgM. Recent findings revealed increased plasma PIGR levels in subjects with subclinical atherosclerosis, although its function remains uncertain.

**Purpose:**

To assess the role of PIGR in atherosclerosis.

**Methods:**

We analyzed PIGR levels in human atherosclerotic plaques compared to healthy aortic samples, as well as in the serum of subjects with peripheral arterial disease (PAD) and controls. Next, we studied the effect of germline *Pigr* deficiency in experimental atherosclerosis (*Ldlr^−/−^/Pigr^−/−^* mice fed a western-diet for 10 weeks). Circulating IgA and IgM levels, as well as B and T cell numbers in spleen and Peyer’s patches (PP), were analyzed by ELISA and flow cytometry, respectively.

**Results:**

PIGR levels were increased in the intima of early human atherosclerotic lesions and in patients with PAD, compared to controls. *Ldlr^−/−^/Pigr^−/−^* mice showed elevated serum IgA and IgM levels, along with an increased number of germinal center B cells in both the spleen and PP. Moreover, *Ldlr^−/−^/Pigr^−/−^* mice displayed a significantly reduced plaque size in the aortic sinus and a strong decrease in foam cells (CD68^+^), while no changes were observed in contractile smooth muscle cells (α-actin^+^) and collagen content compared to control *Ldlr*^−/−^ mice.

**Conclusions:**

Global *Pigr* deficiency decreases atherosclerosis, suggesting that PIGR blockade may have beneficial effects in vascular pathologies.

## Introduction

Cardiovascular (CV) diseases are the leading cause of global mortality and a major contributor to disability worldwide (1). Atherosclerosis is the most common underlying pathology of CV diseases. Several risk factors have been linked to the development of atherosclerosis, including both modifiable (e.g. hypercholesterolemia, obesity, hypertension or smoking) and non-modifiable (e.g. age, sex and family history) factors. Most therapeutic strategies aim to reduce these modifiable risk factors, with a primary focus on hypercholesterolemia. However, the vast majority of patients at risk of atherosclerotic CV disease receive inadequate treatment, leaving them vulnerable to disease progression and acute CV events (2). Furthermore, even among treated patients, there is a significant residual risk (defined as the occurrence of severe CV events during treatment), which highlights the importance of addressing additional novel mechanisms to combat this global pandemic (3, 4).

The pathogenesis of atherosclerosis is characterized by the retention of circulating lipoproteins in the subendothelial space, which leads to vascular remodeling involving resident cells [endothelial cells, vascular smooth muscle cells (VSMCs) and fibroblasts], as well as innate and adaptive immune cells (macrophages, dendritic cells, T and B lymphocytes) (5, 6). The first step in this pathological process involves endothelial dysfunction, which increases the retention of various systemic molecules, including lipoproteins (mainly low-density lipoproteins, LDL). The uptake of LDL (in either its native or oxidized form) by macrophages and VSMCs leads to the formation of foam cells, which are the main component of initial atherosclerotic lesions known as fatty streaks (FS). The progression of FS to more advanced fibrolipidic (FL) plaques is associated with the migration of VSMCs from the media to the intima, where they switch their phenotype from contractile to synthetic. This process promotes the production of collagen and other extracellular matrix (ECM) components. Ultimately, ECM degradation can result in the rupture of atherosclerotic plaques, leading to CV events due to thrombus formation and arterial occlusion.

In addition to innate immune cells, adaptive immune cells play a key role in atherosclerosis. T cells, for instance, are present in atherosclerotic plaques and can differentiate into distinct T helper (Th) or regulatory T (Treg) cell subtypes. These subtypes can either activate or dampen, respectively, the inflammatory response of other immune or tissue-resident cells, support B cells in producing high-affinity antibodies, or exert cytolytic activity. Th1 cells are generally regarded as drivers of atherogenesis, whereas Treg cells are thought to counteract disease progression. Nonetheless, the contributions of other T-cell subsets remain insufficiently characterized (7). B cells are also present in atherosclerotic plaques, albeit to a much lesser extent (8, 9), while plaque-associated tertiary lymphoid organs (TLOs) harbor large numbers of B cells with expanded and diversified B cell receptors (10, 11). B cells are classified into two lineages: B1 cells, which are further subdivided into B1a and B1b subsets, and B2 cells, which comprise marginal zone B cells and follicular B cells (12). Antigen stimulation of B2 cells promote the formation of germinal centers (GC), where B cells proliferate and undergo antibody diversification, ultimately giving rise to long-lived memory B cells or high-affinity plasma cells, which are responsible for antibody secretion (13). B1 cells are considered atheroprotective in mice, possibly through their production of IgM antibodies, which can block the uptake of oxLDL by macrophages in atherosclerotic lesions (14). However, the role of B2 cells in atherosclerosis is more complex, for which both pro-atherogenic (15) and atheroprotective functions (16–19) have been reported, possibly reflecting distinct functional impact of different antigen specificities (20).

IgA is the most abundant immunoglobulin in the human body. It is primarily expressed on mucosal surfaces, where it is released as secretory IgA. While most IgA is generated in gut-associated lymphoid tissues, IgA-producing plasma cells have been found in various pathological tissues, including the vasculature (21). While the roles of IgM, IgG and IgE have been described in atherosclerosis (8, 22), the role of IgA is poorly understood. The limited knowledge of the role of IgA in vascular pathologies may be explained by the differences between mice and humans. In mice, serum IgA is mainly polymeric, whereas in humans it is mostly monomeric. Besides, mice lack homologues of the human Fc alpha receptor (FcαRI) (23). Therefore, other IgA receptors, such as the transferrin receptor, the asialoglycoprotein receptor, and the polymeric immunoglobulin receptor (PIGR), must mediate IgA functions. In particular, PIGR is involved in the transport of polymeric IgA and IgM from the lamina propria to mucosal surfaces across the epithelial barrier (24). However, unlike humans, mice express significant levels of PIGR in their hepatocytes, which results in highly efficient transport of polymeric IgA from the circulation into the bile and thereafter into the intestinal secretions (25). PIGR expression has been primarily observed in intestinal epithelial cells (IECs) and steady-state levels of *Pigr* mRNA in mouse and human IECs are very high (26); however, PIGR has also been detected in the epithelial cells of the lung, liver, and kidneys, as well as in cancer cells of different tissue origins (27). Two recent studies have described increased PIGR and IgA expression in the aorta in a mouse model of vasculitis (28) and in human abdominal aortic aneurysm (29). Very recently, IgM-PIGR interaction in endothelial cells promoted endothelial activation and platelet recruitment (30). However, several PIGR functions are independently of its binding to IgA or IgM and can be instead mediated by its cleaved extracellular portion (secretory component, SC) or the presence of the receptor on cell surfaces (31). In this respect, IL-8 may be solubilized and inactivated by binding to SC (32). Furthermore, PIGR can potential act as a receptor for *Pneumococcus* entry into the brain (33). Recent studies have established a potential role of PIGR in immune-inflammatory diseases, where it can exert both harmful or protective effects (34–36), which in some cases seem to be independent of IgM/IgA interaction.

We previously showed that increased PIGR plasma levels are associated to subclinical atherosclerosis (37). However, whether PIGR contributes to atherosclerosis has not been previously addressed. In the present paper, we analyzed PIGR levels in human atherosclerosis and evaluated the effect of germline *Pigr* deficiency in a hypercholesterolemic mouse model of atherosclerosis.

## Materials and methods

### Human atherosclerotic tissues

Abdominal aortas were collected from brain-deceased organ donors during organ removal for therapeutic transplantation (kidney or liver transplantation) under the authorization of the French Biomedicine Agency (PFS 09-007, BBMRI network, BB-0033-00029). Ethical committee advice and patient written informed consent were obtained before participation (RESAA and AMETHYST studies, CPP Paris-Cochin 2095, 1930, and 1931, INSERM Institutional Review Board, IRB0000388). All human studies conformed to the principles outlined in the Declaration of Helsinki. The aortic tissue was washed and preserved in Ringer’s lactate solution at 4 °C until use. After macroscopic examination by a trained pathologist, the aortas were classified into control aortas (devoid of atheromatous lesions) and aortas with atherosclerotic plaques as previously described (38). In both control and atherosclerotic aortas, the adventitia was removed, followed by separation of the intima and media only in fatty streaks (FS) and fibrolipidic (FL) plaques (as in the healthy aorta, the intima was too thin to be separated from the media). Atherosclerotic plaques as well as control aortas were either incubated in culture medium to obtain tissue-conditioned media or placed in liquid N_2_ for later protein extraction.

For tissue-conditioned media, samples were cut into small pieces (5 mm^2^) and incubated in protein-free RPMI 1640 medium containing antibiotics/antimycotic (Gibco) for 24 hours at 37 °C (6 mL/g of wet tissue). The conditioned media (supernatants containing proteins released by the tissue samples) were obtained after centrifugation (3000 g, 10 min, 20 °C) and kept at -80 °C until further processing.

### Plasma from atherosclerotic subjects

The study was conducted according to the guidelines of the Declaration of Helsinki and approved by the Regional Scientific Ethics Committee of the Region of Central Denmark (M20080028). The cohort was created as a random sample of the cases having peripheral arterial disease (PAD) diagnosed by screening in the Viborg Vascular (VIVA) randomised screening trial as described in the trial protocol (39). In the VIVA trial, the upper limb with the highest blood pressure (BP) was used as a reference. Brachial and ankle pressures were recorded simultaneously, with ankle pressure calculated as the mean of two pedal artery measurements, repeated on the opposite leg.

### Experimental model

All mice were housed either at the CNIC or IIS-FJD animal facility under barrier conditions and a 12 h dark/light cycle with food and water *ad libitum*. All animal procedures were approved by the CNIC and IIS-FJD Ethics Committee and the Madrid regional authorities (PROEX 377/15 and PROEX 238.6/21) and conformed to EU Directive 2010/63/EU and Recommendation 2007/526/EC regarding the protection of animals used for experimental and other scientific purposes, enforced in Spanish law under Real Decreto 1201/2005.

*In vitro* fertilization (IVF) between a male *Pigr^−/−^* mouse (30988, MMRRC) and a female *Ldlr^−/−^* mouse (both on the C57BL/6 background) was performed, and the progeny bred were crossed back to *Ldlr^−/−^* mice for 5 generations to obtain the double knockout (*Ldlr^−/−^ Pigr^−/−^*) and their littermate control (*Ldlr^−/−^ Pigr^+/+^*) mice. To study the effect of *Pigr* deletion on atherosclerotic lesions, 10-week-old *Ldlr^−/−^Pigr^−/−^* (n=14 male and n=8 females) and *Ldlr^−/−^Pigr^+/+^* (n=8 males and n=13 females) were containing 22.1% of fat and 0.21% cholesterol (EF D12079 mod, Ssniff Spezialdiäten) for 10 weeks. At the end of the study, after a 16-h fast, mice were rendered deeply anesthetized with ketamine (100 mg/kg) and xylazine (15 mg/kg) (intraperitoneal) and euthanasia was completed by exsanguination via cardiac puncture, with death confirmed prior to tissue collection. Blood samples were collected for biochemistry. Total serum cholesterol, HDL-c, and triglycerides were measured enzymatically using commercial kits adapted for a COBAS 6000 autoanalyzer (Roche Diagnostics).

### Morphometric analysis

Hearts containing aortic roots were carefully dissected and frozen in optimal cutting temperature (OCT) compound. Aortic roots were sectioned at 5 µm thickness beginning proximally at the first evidence of the aortic valves at their attachment site of aorta until the disappearance of valve cusps. Sections were treated with oil red O (ORO)/hematoxylin for lipid staining at 100-µm intervals from 0 to the end of the aortic valves, and 8 slides per mice were analyzed. Maximal lesion area was calculated for each mouse by averaging the values for three sections. The individual maximal lesion areas were further averaged to determine the maximal lesion area for each mouse group. The volume of atherosclerotic lesions was estimated by calculating the area under the curve (AUC) for each condition. Lipid content was defined as the percentage of ORO staining area from total plaque area. Picrosirius red staining was performed for analysis of collagen content by measuring birefringence to plane-polarized light. Images were taking using a Leica DMD108 Microscope. Computer-assisted morphometric analysis was performed with Image J software and the Image-Pro Plus software.

### Immunofluorescence

Frozen sections were fixed in 10% formalin for 7 min and then washed under running water for 7 min prior to blocking endogenous peroxidase and primary antibody incubation [anti-mouse CD68 (ab53444, Abcam, 1:200) and anti-mouse alpha-smooth muscle actin (α-SMA) Cy3 conjugated (C6198, Sigma, 1:1000)]. After the incubation for 1 hour away from light with species-specific secondary fluorescent conjugated antibodies, nuclei were stained with 4′,6-diamidino-2-phenylindole (DAPI) and slides mounted with fluorsave medium. Alexa Fluor 488 donkey anti-rat IgG (Invitrogen) was used as secondary antibody. Stained tissues were examined using fluorescence microscopy and images were obtained using a Zeiss microscope. Computer-assisted morphometric analysis was performed with Image J software and the Image-Pro Plus software. The threshold setting for area measurement was equal for all images. The adjacent slides to the maximal lesion from each animal were examined in a blinded manner. Results were expressed as % positive area versus total area (CD68 and α-SMA).

### Protein extraction and immunoblotting

Human atherosclerotic tissues, as well as aortic wall tissues from healthy human donors, were collected and incubated with lysis buffer containing 10 mM Tris-HCl pH 7.4 buffer, 150 mM NaCl, 0.5% NP-40, 1% Triton X-100, 1 mM EDTA, 1 mM EGTA, 10 mM NaF, 1 mM DTT, 1 mM PMSF and protease and phosphatase inhibitors and pelleted. After determining protein using the Pierce BCA Protein Assay Kit (Thermo Scientific), equal amounts of proteins (20 µg) were subjected to sodium dodecyl sulphate (SDS) polyacrylamide gel electrophoresis and then transferred to a nitrocellulose membrane (Bio-Rad). After blocking with 5% BSA in TBS containing 0.1% Tween-20, blots were incubated overnight with anti-human PIGR (1:1000, AF2717, R&D) or anti-human GAPDH (1:10000, MAB374, Sigma) at 4 °C. Blots were then washed and incubated with the appropriate horseradish peroxidase (HRP)-conjugated secondary antibody for one hour and visualized using the ECL substrate kit (Thermo Scientific). Membranes were subjected to densitometry (Image J), and values were normalized against GAPDH.

### Enzyme-linked immunosorbent assay

PIGR levels in the tissue-conditioned media of human atherosclerotic plaques walls and control aortas, as well as in human plasma samples, were measured according to the manufacturer’s protocol (ab282302, Abcam). In short, plates were coated with samples or standards, followed by the antibody cocktail (capture and detector antibodies). After incubation at room temperature for 1 hour, wells were washed to remove unbound material and TMB Development Solution was added. The optical density at 450 nm (OD_450_) was measured. After factoring sample dilutions, PIGR concentrations in the original samples were finally calculated.

IgA and IgM levels were analyzed in human plasma samples following manufacturer´s protocol (BMS2096 and BMS2098, Invitrogen). Total mouse IgA and IgM, and ox-LDL specific antibody titers in serum of mice were determined using mouse IgA and mouse IgM ELISA Quantitation Kits (Bethyl Laboratories; E99–103 and E90-101, respectively) used in accordance with the manufacturer’s instructions.

In brief, plates were coated with goat anti-mouse IgM capture antibodies or ox-LDL (3 µg/ml) overnight at 4 °C for IgM ELISA. For IgA ELISA, an anti-mouse IgA antibody was already pre-adsorbed on the surface of microtiter wells. Then, plates were blocked and incubated with diluted samples or standards, followed by incubation with anti-mouse IgA or IgM detection antibodies conjugated to HRP. TMB substrate solution was subsequently added and the OD_450_ was measured. IgA and IgM concentrations in the original samples were finally calculated.

### Flow cytometry

Spleens and Peyer’s Patches were harvested and placed in cold PBS supplemented with 2% Fetal Bovine Serum (FBS, Sigma-Aldrich, F7524-1654682). Spleens were mechanically dissociated through a 70 µm cell strainer. Red blood cells were lysed using ACK Lysis Buffer (Lonza, 10-548E) for 4 minutes at room temperature, followed by washing and resuspension in staining buffer (PBS + 2% FBS + 2mM EDTA) for downstream flow cytometry analysis. Peyer’s Patches were mechanically dissociated, washed and resuspended for downstream flow cytometry analysis.

Single-cell suspensions were blocked with anti-mouse CD16/CD32 antibodies (1/50) and stained with combinations of the following fluorochrome-conjugated or biotin-conjugated antibodies: CD19-APCeF780 (1D3, 1/100, eBioscience), CD3-BV711 (145-2C11, 1/100, BD Biosciences), CD11b-A647 (M1/70, 1/100, BD Biosciences), F4/80-PECy7 (BM8, 1/100, Biolegend), Ly6G-PE (1A8, 1/100, BD Biosciences), Ly6C-FITC (AL-21, 1/100, BD Biosciences), B220-BV421 (RA3-6B2, 1/1000, BD Horizon), B220-PE (RA3-6B2, 1/200, BD Pharmingen), GL7-FITC (GL7, 1/400, BD Pharmingen), Fas-PECy7 (Jo2, 1/100, BD Pharmingen), IgA-biotin (polyclonal IgG, 1/200, AbD Serotec), PDL2-APC (25, 1/100, BD Biosciences), CD4-PECy7 (GK1.5, 1/200, Tonbo Biosciences), CXCR5- APC (L138D7, 1/100, Biolegend), PD1- BV421 (29F.1A12, 1/100, Biolegend), CD25-biotin (7D4, 1/100, BD Pharmingen), CD19-PE (1D3, 1/200, BD Pharmingen), IgM-APC (II/41, 1/100, BD Pharmingen), CD43-biotin (S7, 1/200, BD Pharmingen), CD5-FITC (53-7.3, 1/100, BD Pharmingen), FoxP3-AF488 (MF23, 1/100, BD Biosciences), F4/80- PECy7 (1/200, Invitrogen) and Ly6C-FITC (1/200, BD Pharmingen). Streptavidin ST-APC, PE, PECy7 or BV421 were used in the case of biotin-conjugated antibodies. Only live lymphocytes were analyzed (LIVE/DEAD^®^ Fixable Yellow Dead Cell Stain Kit, Thermo Fisher, L34967). For FoxP3 intracellular staining, cells were fixated and permeabilized using the Foxp3/Transcription Factor Staining Buffer Set (Thermo Fisher Scientific). Samples were acquired on LSRFortessa software v6.2 Flow Cytometer (BD Biosciences) using FACSDiva software v9.3.1 and analyzed with FlowJo V10.10.

### Statistical analysis

Data are presented as mean ± standard error of the mean (SEM). For the analysis of data from human atherosclerotic tissue samples, groups were compared using the non-parametric Kruskal–Wallis test followed by Dunn’s *post hoc* test. For analysis of human plasma data, ankle braquial index (ABI) measurements needed log transformation to achieve a normal distribution. The association between PIGR and ABI was studied by Pearson´s correlation analysis. To evaluate the independent association of PIGR with ABI, a multivariate linear regression analysis was performed adjusted by known confounding factors [age, family disposition to PAD (1st degree relative), smoke, height, weight, hypertension, diabetes mellitus, and previous CVD]. For the analysis of data from the experimental models, groups were compared using the non-parametric Mann–Whitney U test. Statistical analyses were performed using GraphPad Prism software (version 8.0.2 for Windows, GraphPad Software), with statistical significance set at p < 0.05 (*p < 0.05, **p < 0.01, ***p < 0.001 and ****p < 0.0001).

## Results

### PIGR expression is increased in human atherosclerosis

**Figure 1:**
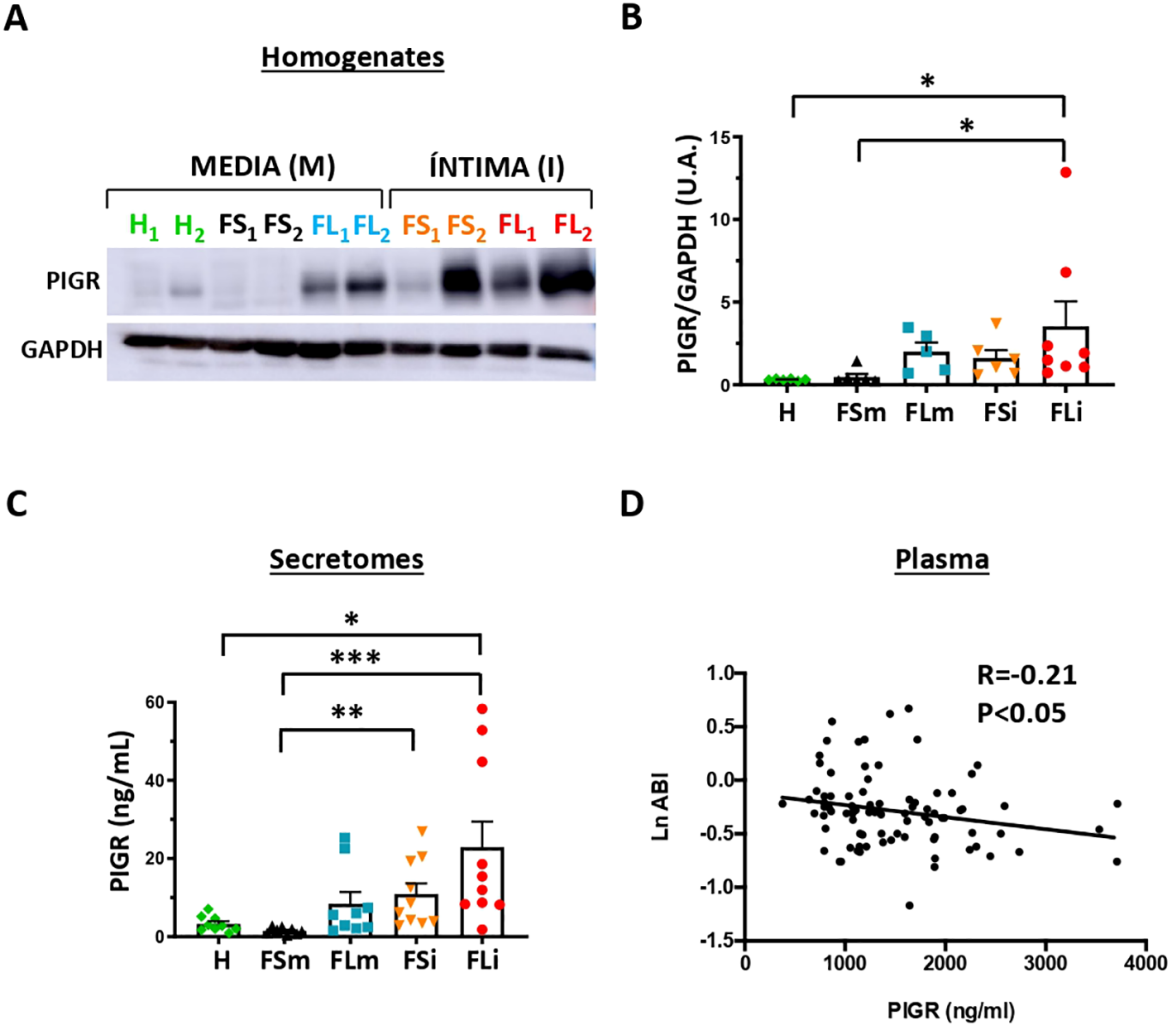
PIGR in human atherosclerosis. Western blot **(A)** and densitometric analysis **(B)** of PIGR in early atherosclerotic plaques (FS media n=6; FL media n=5; FS intima n=6; FL intima n=8) and healthy aortas (H, n=6). Data represent means ± SEM. Kruskal-Wallis with Dunn’s comparison test for multiple comparisons. *p<0,05. FS, fatty streak. FL, fibrolipidic lesion **(C)**. Quantification by ELISA of PIGR concentration in the secretomes of early atherosclerotic plaques (FS media n=8; FL media n=9; FS intima n=10; FL intima n=10) and healthy aortic tissues (H, n=9). Data represent means ± SEM. Kruskal-Wallis with Dunn’s comparison test for multiple comparisons. *p<0,05, **p<0,01, ***p<0,001. **(D)**. Correlation of PIGR concentration in plasma with logarithmic ABI in PAD patients (n=86, Pearson correlation test, r=-0.21, p<0.05). ABI, ankle brakial index.

To study the involvement of PIGR in human atherosclerosis, we first analyzed PIGR protein expression in early human atherosclerotic plaques (fatty streaks, FS, and fibrolipidic lesion, FL) compared with healthy (H) aortic wall samples by Western blot. We found that PIGR was significantly increased in the intima of human atherosclerotic lesions (**Figures 1A, B**, **Supplementary Figure 1**). As PIGR can be proteolyzed and secreted, we analyzed PIGR levels in the tissue-conditioned media (secretomes) of early human atherosclerotic plaques and healthy aortic wall samples by ELISA. PIGR levels were also significantly increased in the secretome of the intima of human atherosclerotic lesions (**Figure 1C**).

PIGR plasma concentration was analyzed in a group of 86 atherosclerotic patients with peripheral arterial disease (PAD) to test the potential association with disease progression. For that purpose, we analyzed a surrogate marker of PAD severity, the ankle braquial index (ABI) whose lower values reflects higher atherosclerosis and blood-flow obstructions in the leg (40). PIGR plasma concentration negatively correlated with ABI in PAD patients (rho=-0.21, p=0.44, **Figure 1D**), indicating the direct association of higher PIGR plasma concentration with PAD severity. In contrast, IgA and IgM did not correlate with ABI in PAD patients (IgA=0.021 and IgM=0.102, p>0.05). Among different risk factors, smoking was also significantly inversely associated to ABI (**Table 1**). Multivariate linear regression analysis demonstrated that the association of PIGR with ABI was independent of other risk factors (**Table 1**). Thus, all these findings suggest a potential contribution of PIGR in the development of atherosclerosis.

**Table 1:** Multivariate linear regression analysis with ABI as dependent variable.

Variable	Beta	Sig.
PIGR	-0.212	0.044
Age	-0.086	0.425
Height	0.121	0.293
Weight	0.042	0.710
Familial history of PAD	0.100	0.336
Smoke	-0.273	0.017
Diabetes Mellitus	0.048	0.678
Hypertension	-0.107	0.345
Previous CV disease	0.155	0.169

### Germline *Pigr* deletion decreases atherosclerosis in a hyperlipidemic model mice

**Figure 2:**
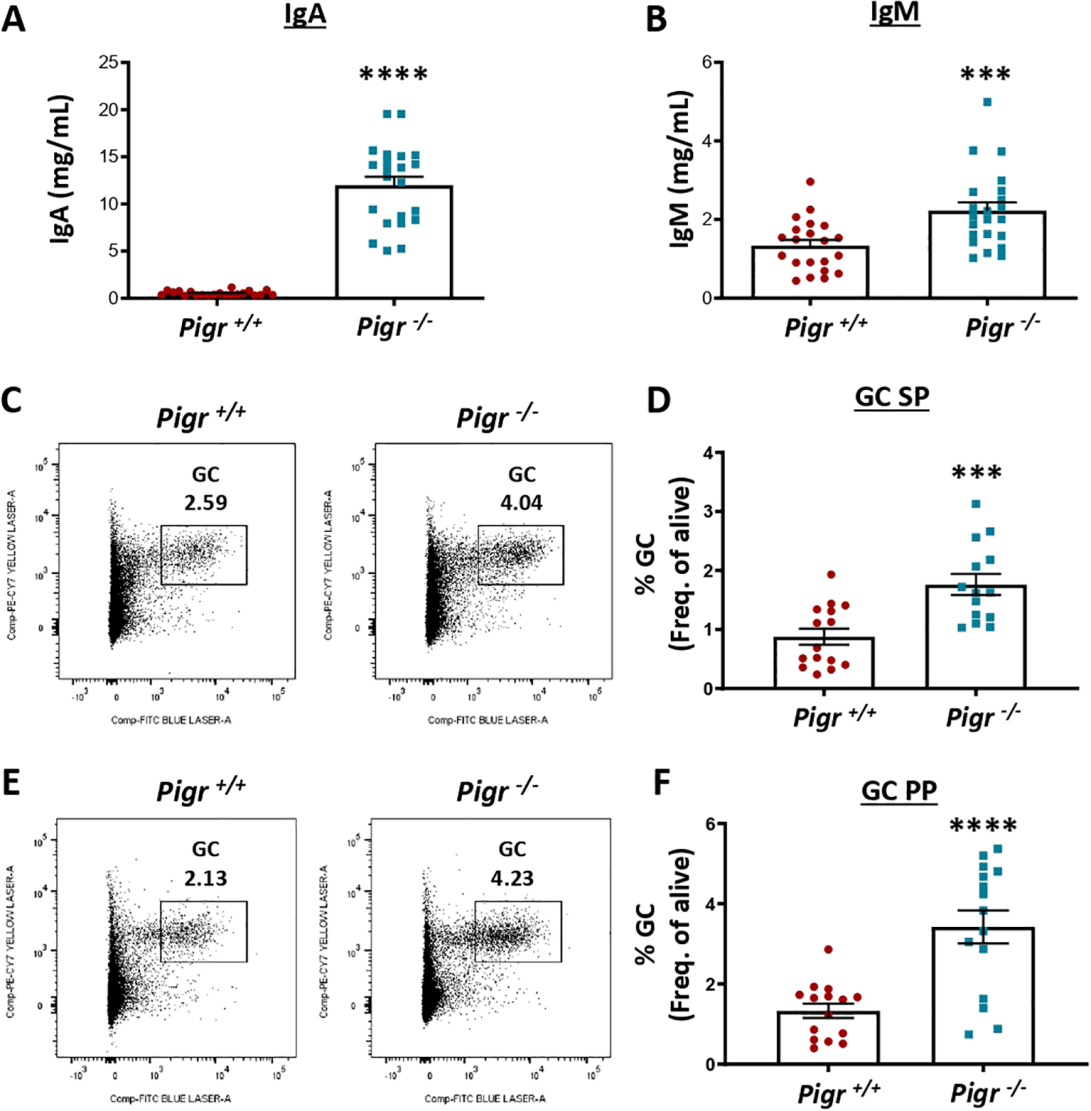
Germinal center response in *Pigr* deficient mice under hypercholesterolemic conditions. Quantification by ELISA of IgA **(A)** and IgM **(B)** levels in serum from *Ldlr^−/−^ Pigr^−/−^* (n=22) and *Ldlr^−/−^ Pigr^+/+^* (n=21) mice subjected to 10 weeks of western-diet. Representative FACS plots and quantification of GC B cells in spleen **(C, D)** and Peyer´s patches **(E, F)** from *Ldlr^−/−^ Pigr^−/−^* (n=15) and *Ldlr^−/−^ Pigr^+/+^* (n=14-15). Each dot in the graphs represents a biological replicate (individual mouse). Population percentages in FACS graphs indicate the frequency of live cells. Mann-Whitney U test. ***p < 0.001 and ****p < 0.0001.

**Figure 3:**
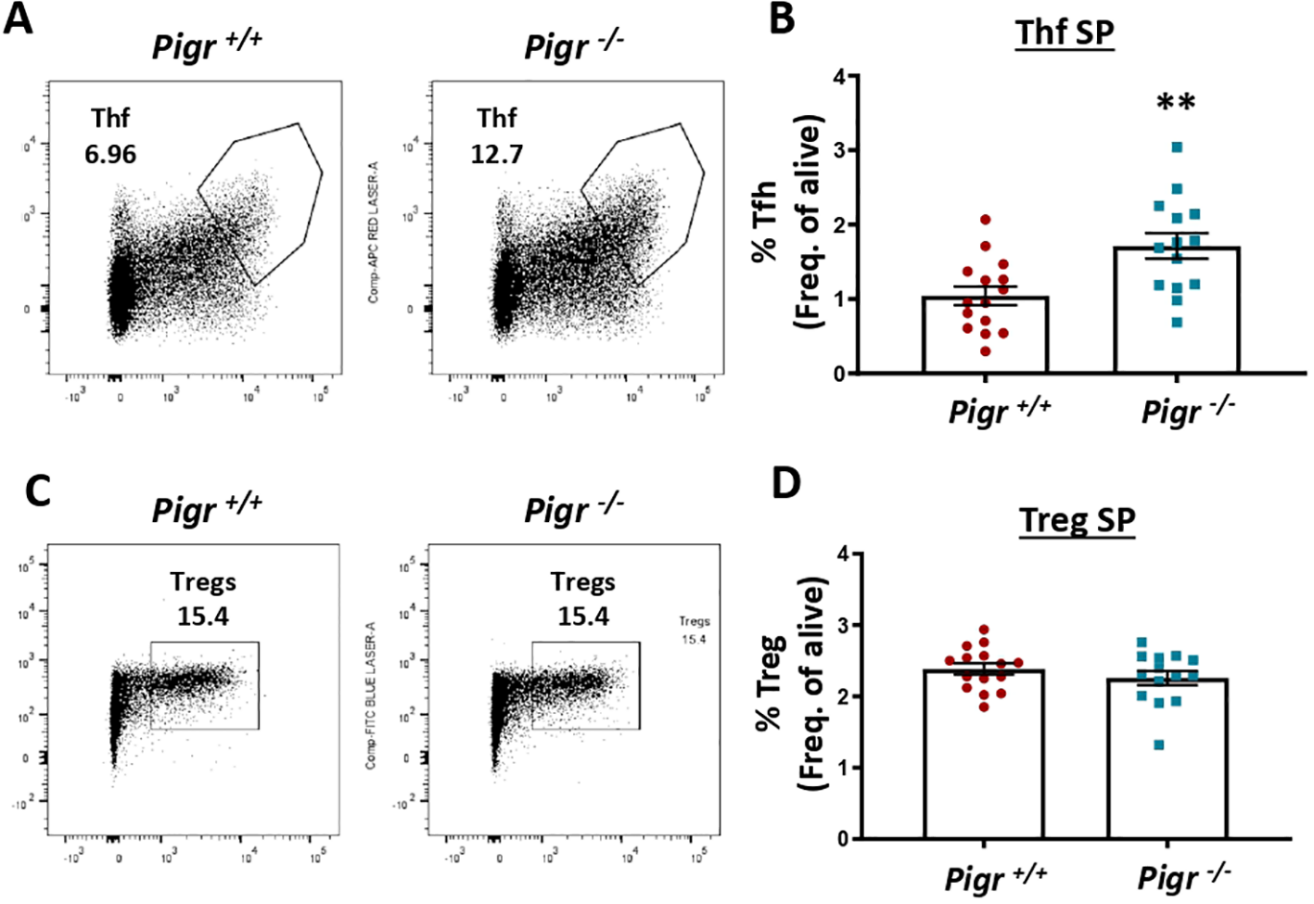
TfH and Tregs in spleen of *Pigr* deficient mice under hypercholesterolemic conditions. Representative FACS plots and quantification of TfH cells **(A, B)** and Tregs **(C, D)** from *Ldlr^−/−^ Pigr^−/−^* (n=14-15) and *Ldlr^−/−^ Pigr^+/+^* (n=14-15). Each dot in the graphs represents a biological replicate (individual mouse). Population percentages in FACS graphs indicate the frequency of live cells. Mann-Whitney U test. **p < 0.01.

To assess whether PIGR could contribute to the development of atherosclerosis *in vivo*, we evaluated the effect of global *Pigr* deficiency in mice lacking both *Pigr* and *Ldlr* (*Ldlr^−/−^ Pigr^−/−^*) and their littermate controls (*Ldlr^−/−^ Pigr^+/+^* single knockout mice) for 10 weeks to induce atherosclerosis. No differences were observed in body weight (not shown) nor in serum lipids (**Supplementary Figure 2**) between both genotypes at the end of the follow-up period. As the main known function of PIGR is the transport of polymeric Ig through the intestinal epithelium, we first measured the IgA and IgM concentration in mouse serum. We observed a significant increase of IgA (>20 fold) and IgM (>1.5 fold) in *Ldlr^−/−^ Pigr^−/−^* mice compared to their littermate controls (**Figures 2A, B**). However, no significant differences were observed in IgM anti-oxLDL antibody titers between *Ldlr^−/−^ Pigr^−/−^* and *Ldlr^−/−^ Pigr^+/+^* mice (0.19±0.02 vs 0.16±0.01 absorbance units, p=0.1; **Supplementary Figure 3**), while IgA anti-oxLDL signal was negligible. Interestingly, both, the spleen (**Figures 2C, D**) and the Peyer´s patches (PP) (**Figures 2E, F**) showed a significant increase of GC B-cells in *Ldlr^−/−^ Pigr^−/−^* mice compared to their controls. Accordingly, follicular helper T (Tfh) cells were increased in the spleen of *Ldlr^−/−^ Pigr^−/−^* (**Figures 3A, B**), while no changes were detected in Treg (CD25/FoxP3) (**Figures 3C, D**). No significant differences in total splenic cells were observed in *Ldlr^−/−^ Pigr^−/−^*, while total frequency of T-cells (CD3^+^) was decreased and concomitantly, CD19^+^ B cells were proportionally increased (**Supplementary Figure 4**). Moreover, no differences in spleen monocytes or macrophages were observed (**Supplementary Figure 5**). Thus, PIGR deficiency associates with increased IgA and IgM titers in sera and an enhanced GC response.

**Figure 4:**
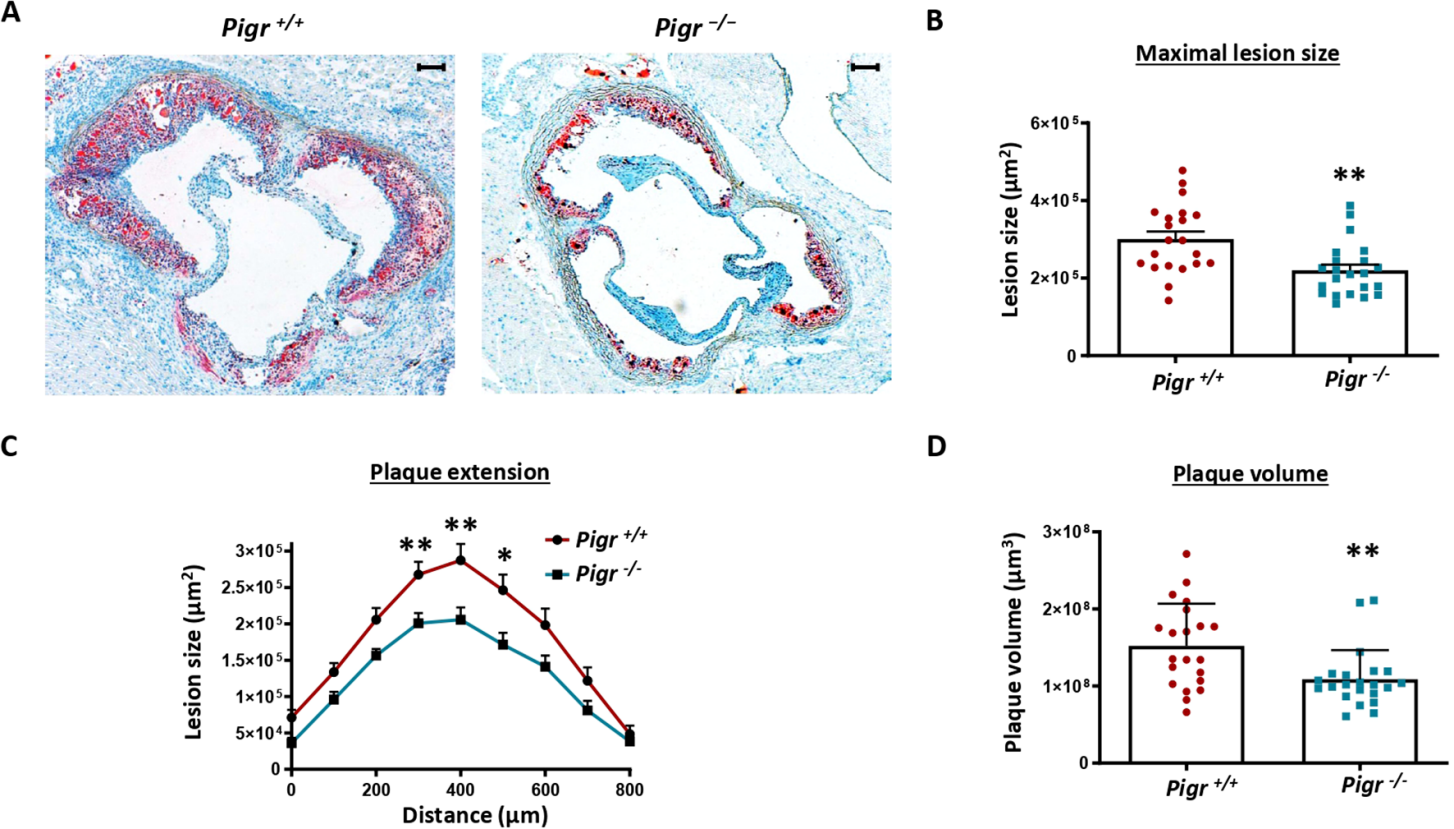
Representative photographs **(A)** and quantification of lesion area (μm^2^), **(B)**, maximum atherosclerotic lesion area (μm^2^) as a function of distance (μm) **(C)**, and area under the curve (AUC) values, representing total plaque volume (μm3) **(D)**, in the aortic root from *Ldlr*−/− *Pigr*−/− (n=22) and *Ldlr*−/− *Pigr*+/+ (n=21) mice. Data represent means ± SEM. Mann-Whitney U test. *p<0,05, **p<0,01. Scale bars, 100 µm.

**Figure 5:**
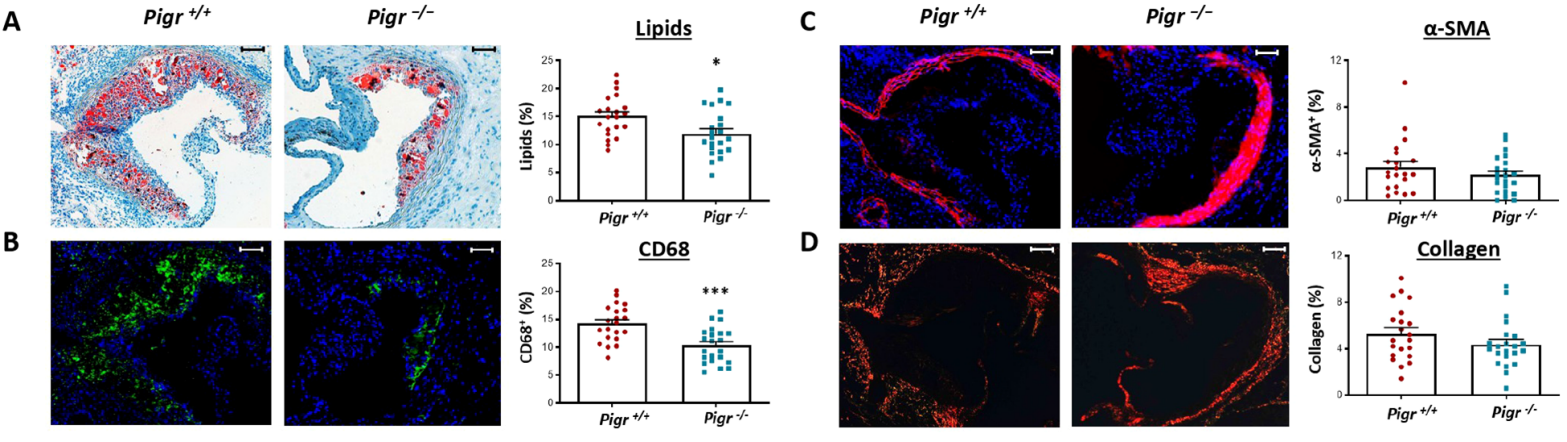
*Pigr* deficiency decreases intralesional lipids and macrophage infiltration in *Ldlr* knockout mice. Representative photographs and quantification of ORO **(A)**, CD68 **(B)**, α-SMA **(C)** and collagen **(D)** in the aortic root from *Ldlr^−/−^ Pigr^−/−^* (n=22) and *Ldlr^−/−^ Pigr^+/+^* (n=21) mice. Data represent means ± SEM. Mann-Whitney U test. *p<0,05, ***p<0,001. Scale bars, 100 µm.

To assess the impact of PIGR deficiency on atherosclerosis progression, we quantified atherosclerotic lesion size in the aortic sinus. We found that atherosclerosis lesion was significantly reduced in *Ldlr^−/−^ Pigr^−/−^* mice compared to *Ldlr^−/−^ Pigr^+/+^* mice (**Figures 4A, B**). Likewise, analysis of atherosclerotic plaque volume in aortic sinus lesions revealed a significant reduction in *Ldlr^−/−^ Pigr^−/−^* mice compared to controls (**Figures 4C, D**). Since plaque composition is crucial for the progression of atherosclerosis, we evaluated lipid content, CD68 and α-SMA expression as well as collagen content in atherosclerotic plaques. Lipid content and CD68 expression were significantly decreased in mice lacking *Pigr* compared to controls (**Figures 5A, B**). However, no statistically significant differences were detected between genotypes in either α-SMA expression or collagen content (**Figures 5C, D**). Analysis by gender showed overall similar results (**Supplementary Figures 6**-**8**). Thus, our data suggests that PIGR deficiency is atheroprotective.

## Discussion

**Figure 6:**
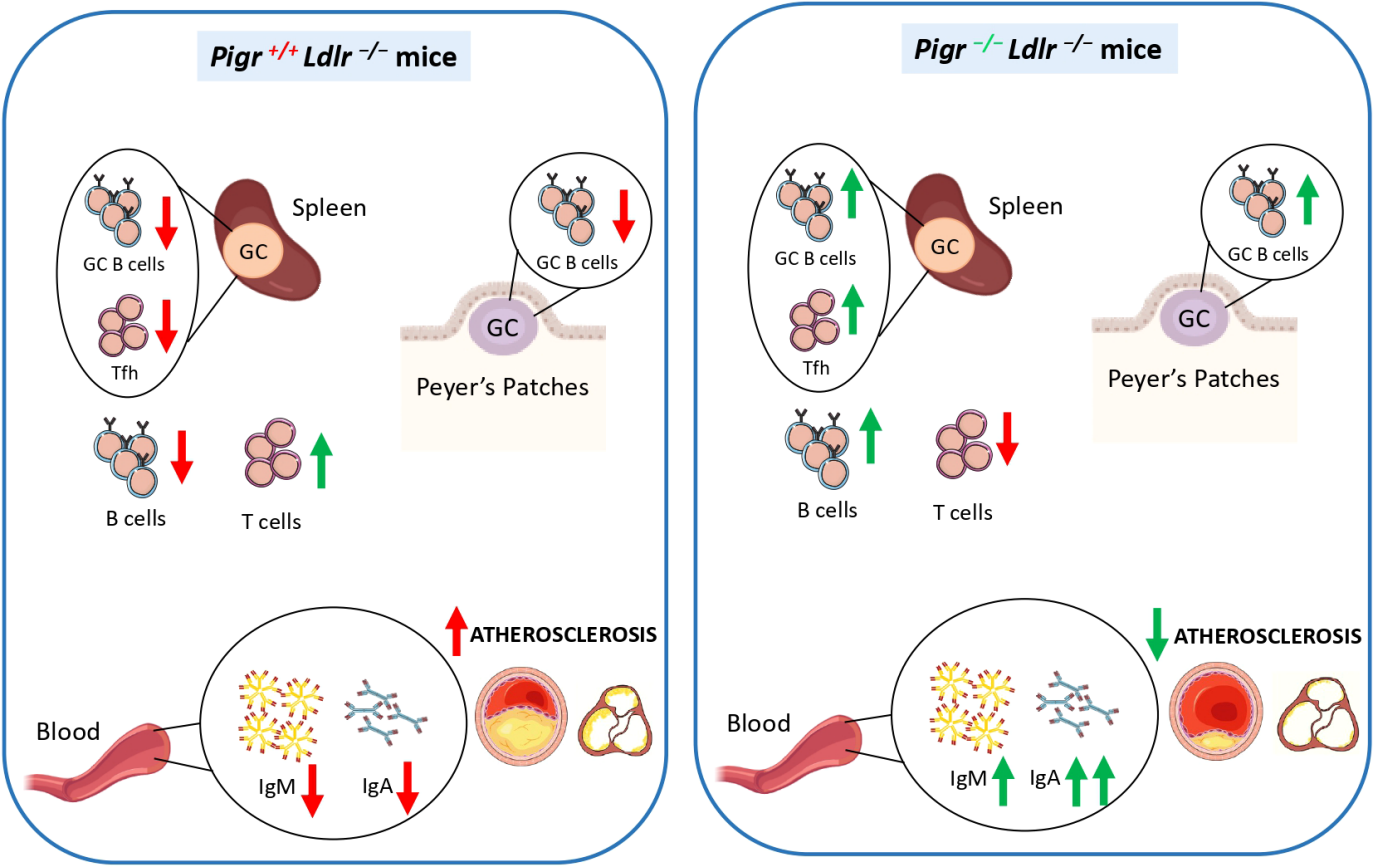
Summary of the main findings obtained in the present study.

In the present paper, we described increased levels of PIGR in atherosclerotic tissues and a positive correlation between PIGR plasma levels and atherossclerosis severity in human atherosclerotic subjects. Furthermore, we observed that PIGR deletion in atherosclerotic mice leads to elevated systemic IgA and IgM levels, enhances the GC B-cell response, and reduces plaque development and inflammatory infiltration (**Figure 6**).

PIGR expression and secretion are modulated by multiple immunological, microbial, hormonal, and environmental factors (41). A variety of proinflammatory immune mediators, such as IFN-γ and TNF-α, as well as TLR-3 and TLR-4 ligands, which are produced in response to various bacterial and viral infections, play a key role in upregulating PIGR expression. In this work, we have shown a significant increase of PIGR levels in the intima of human atherosclerotic tissue and tissue-secretome as compared to healthy aortic tissues. Therefore, it seems plausible that, under the proinflammatory scenario present in atherosclerosis, PIGR is overexpressed and released. In this respect, we detected a positive association between PIGR and subclinical atherosclerosis in two different cohorts (37). In agreement, previous studies described an association of plasma PIGR levels with myocardial infarction (42), the Framingham coronary heart disease risk scale (43) and with the development of carotid atherosclerosis (44). PIGR was also described as a prognostic biomarker of cardiac events in asymptomatic subjects (45). Similarly, a previous study showed increased levels of IgA in patients with atherosclerosis compared to controls (46). Moreover, IgA, but not IgM, was predictive of future myocardial infarction and cardiac death in men with dyslipidemia (47). In the present study, we observed a correlation of PIGR, but not of IgA and IgM, with ABI in plasma of patients with clinical atherosclerosis, indicating that increasing levels of PIGR are associated to atherosclerosis severity. However, as the correlation coefficient was weak, further studies will be needed to confirm its potential translational value. Interestingly, the association of PIGR with PAD progression was independent of traditional risk factors, suggesting that analysis of PIGR could afford additional information to that used in risk scales used in the clinical practice. All this data supports the implication of PIGR in atherosclerosis progression.

To further explore the potential implication of PIGR in atherosclerosis, we tested the effect of germline deletion of *Pigr* in a hypercholesterolemic model of atherosclerosis. Global deficiency of *Pigr* does not affect systemic lipid levels, but has a remarkable increase in serum IgA in agreement with previous reports (48, 49), which has been primary linked to the absence of the hepatic IgA pump operating in rodents (50). We also observed a slight increase on IgM levels while previous studies did not observe this difference (48, 49). These divergent findings may be explained by differences in the *Pigr^−/−^* mouse lines analyzed or by the incorporation of the *Ldlr^−/−^* atherogenic background in the current experimental setting. In addition, we observed increased GC B-cells in PP and spleen of *Ldlr^−/−^ Pigr^−/−^* mice compared to their controls. Although the origin of the antigens involved in this GC activation remains unknown, it seems plausible that the defective mucosal barrier function in *Pigr*-deficient mice (35) could favor the exposure to exogenous antigens. Alternatively endogenous(self-)/modified(neo-) antigens could also be triggering this immune response as previously reported in atherosclerotic mice and in human atherosclerotic patients (51–53). Regardless of the nature of the antigens involved, the spleen GC response is suggestive of a systemic immune response. On the whole, our data demonstrate that under hypercholesterolemic/atherogenic conditions, *Ldlr^−/−^ Pigr^−/−^* mice exhibit increased systemic IgA and IgM levels along with GC responses. GC and T cell dependent responses have been assigned both atherogenic (17, 54), and atheroprotective roles (18, 20, 22, 55). Our results in *Ldlr^−/−^ Pigr^−/−^* mice indicate that a GC response is compatible with an atheroprotective effect.

The role of IgA in atherosclerosis has not been fully addressed (8, 22). Mice lacking the Fc receptor for IgA and IgM, *Fcamr^−/−^*, display a significant reduction in cardiovascular inflammation in the aortic root, coronary arteries, and the abdominal aorta in a vasculitis model induced by *Lactobacillus casei* cell wall extracts (28). Likewise, blocking IgA actions by using an antibody against Fcα expression decreased atherosclerosis in mice by inhibiting macrophage infiltration (56). Here, we showed that, despite having increased serum levels of IgA, *Pigr* deficiency delayed atherosclerosis progression. These findings suggest that two mechanisms involving the IgA-PIGR axis can be involved in aherosclerosis progression. On one hand, increased IgA levels can promote inflammation through its receptors (57). On the other, PIGR may have additional proinflammatory functions independent of its binding to IgA (36). In the absence of PIGR, the reduction of this inflammatory milieu would protect against atherosclerosis, compensating the presumably milder atherogenic effect of increased IgA levels. Regardless of whether the atheroprotection observed in *Ldlr^−/−^ Pigr^−/−^* mice relies on the interaction with its known (including IgA and IgM) or unknown ligands, our results suggest that local blockade of PIGR within atherosclerotic plaques may represent a potential therapeutic strategy to prevent disease progression. Although the reduction of lesion size and/or volume was limited, it is important to highlight that plaque composition is a key determinant for undesired fatal complications associated to plaque rupture (58). In this regard, plaques in *Ldlr^−/−^ Pigr^−/−^* mice showed lower lipid and macrophage content, so they could be considered as more stable plaques. Interestingly, some studies described protective functions of neutralizing PIGR in different diseases (33, 59, 60). Whether similar strategies would be feasible in atherosclerosis, deserves further studies.

### Limitations

The study has several limitations:

1) The number of patients analyzed are rather low (n=86) and include only male subjects, so future studies in larger cohorts including both genders will be needed to assess the potential predictive role of PIGR in atherosclerosis; 2) The potential translation of the results observed in mice to humans is hindered by several items, including the differences between atherosclerosis in humans and experimental models in mice (61), and the differences in IgA metabolism due to differences in IgA isotypes and receptors (23, 24); 3) Our study is descriptive since the use of global *Pigr* knockout mice presents certain limitations, including the systemic increase in IgA and IgM levels, which may influence the development of atherosclerosis. In addition, the absence of IgA may have effects on the gut and liver that could indirectly impact disease progression. In this respect, future experiments with cell-specific (macrophages/endothelial cells/hepatocytes) deficiency in PIGR would be needed to clarify the specific mechanisms underlying the atheroprotective effects observed in this study.

## Data Availability

The raw data supporting the conclusions of this article will be made available by the authors.

## Supplementary material

Supplementary Figure 1 Uncropped scans of representative immunoblots of PIGR and GAPDH (loading control) in early atherosclerotic plaques.

Supplementary Figure 2 *Pigr* deficiency does not modify circulating lipids in *Ldlr* knockout mice. Serum determination of total-cholesterol **(A)**, triglycerides **(B)**, HDL-cholesterol **(C)** and VLDL/LDL-cholesterol **(D)** from *Ldlr^−/−^ Pigr^−/−^* (n=22) and *Ldlr^−/−^ Pigr^+/+^* (n=21) mice. Values shown are the mean ± SEM.

Supplementary Figure 3 Pigr deficiency does not modify IgM anti-oxLDL antibody levels in mouse serum. Quantification by ELISA of IgM anti-oxLDL antibody levels in serum from *Ldlr^−/−^ Pigr^−/−^* (n=22) and *Ldlr^−/−^ Pigr^+/+^* (n=21) mice subjected to 10 weeks of western-diet.

Supplementary Figure 4 Total cells, T-cells and B-cells in spleen of *Pigr* deficient mice under hypercholesterolemic conditions. Representative FACS plots **(A)** and quantification of total cells **(B)**, T-cells **(C)** and B-cells **(D)** from *Ldlr^−/−^ Pigr^−/−^* (n=15) and *Ldlr^−/−^ Pigr^+/+^* (n=14-15). Each dot in the graphs represents a biological replicate (individual mouse). Population percentages in FACS graphs indicate the frequency of live cells. Mann-Whitney U test.*p<0.05, **p < 0.01.

Supplementary Figure 5 Monocytes and macrophages in spleen of *Pigr* deficient mice under hypercholesterolemic conditions. Representative FACS plots **(A)** and quantification of monocytes **(A)** and macrophages **(B)** from *Ldlr^−/−^ Pigr^−/−^* (n=15) and *Ldlr^−/−^ Pigr^+/+^* (n=14). Each dot in the graphs represents a biological replicate (individual mouse). Population percentages in FACS graphs indicate the frequency of live cells. Mann-Whitney U test.

Supplementary Figure 6 Gender analysis of lesion size **(A)** and plaque volume **(B)** in mice. Male *Ldlr^−/−^ Pigr^−/−^* (n=14 and 8, respectively) and female *Ldlr^−/−^ Pigr^+/+^* (n=8 and n=13, respectively). Mann-Whitney U test. *p<0.05, **p < 0.01.

Supplementary Figure 7 Gender analysis of lipids **(A)** and macrophages **(B)** in atherosclerotic plaques of mice. Male *Ldlr^−/−^ Pigr^−/−^* (n=14 and 8, respectively) and female *Ldlr^−/−^ Pigr^+/+^* (n=8 and n=13, respectively). Mann-Whitney U test.*p<0.05, ***p<0,001.

Supplementary Figure 8 Gender analysis of contractile VSMC **(A)** and collagen **(B)** in atherosclerotic plaques of mice. Male *Ldlr^−/−^ Pigr^−/−^* (n=14 and 8, respectively) and female *Ldlr^−/−^ Pigr^+/+^* (n=8 and n=13, respectively). Mann-Whitney U test.
