# Supplementary material for: Polymeric immunoglobulin receptor deficiency attenuates experimental atherosclerosis
Source: Front Immunol. 2026 Apr 23;17:1774396. doi: 10.3389/fimmu.2026.1774396 (PMC13149079; doi:10.3389/fimmu.2026.1774396)
Supplement: Supplementary Figure 1 — Uncropped scans of representative immunoblots of PIGR and GAPDH (loading control) in early atherosclerotic plaques. [file Presentation1.pptx]

## Slide 1
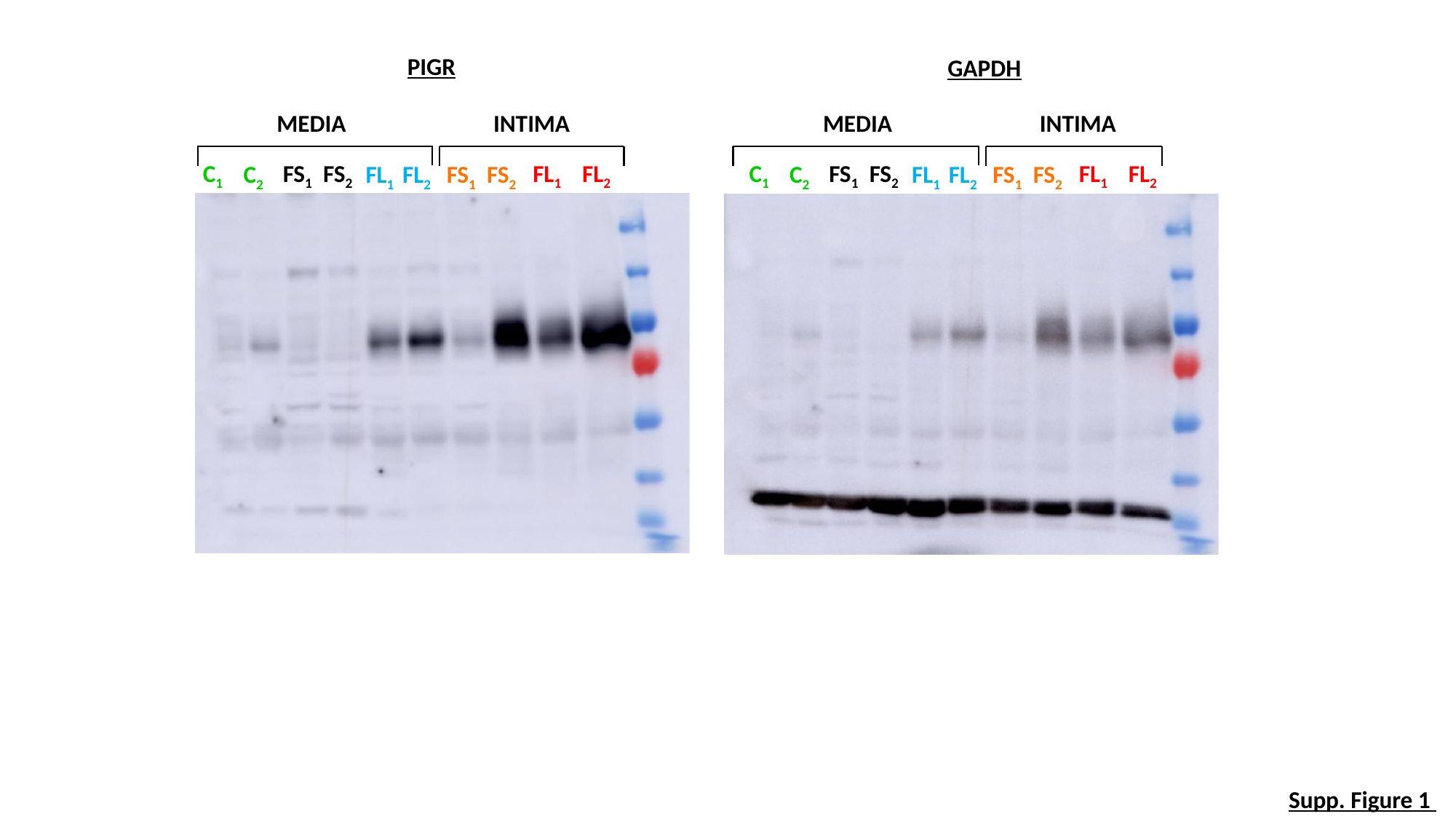

PIGR
GAPDH
MEDIA
INTIMA
MEDIA
INTIMA
C1
FS1
FS2
FL1
FL2
C1
FS1
FS2
FL1
FL2
C2
FL1
FL2
FS1
FS2
C2
FL1
FL2
FS1
FS2
Supp. Figure 1

## Slide 2
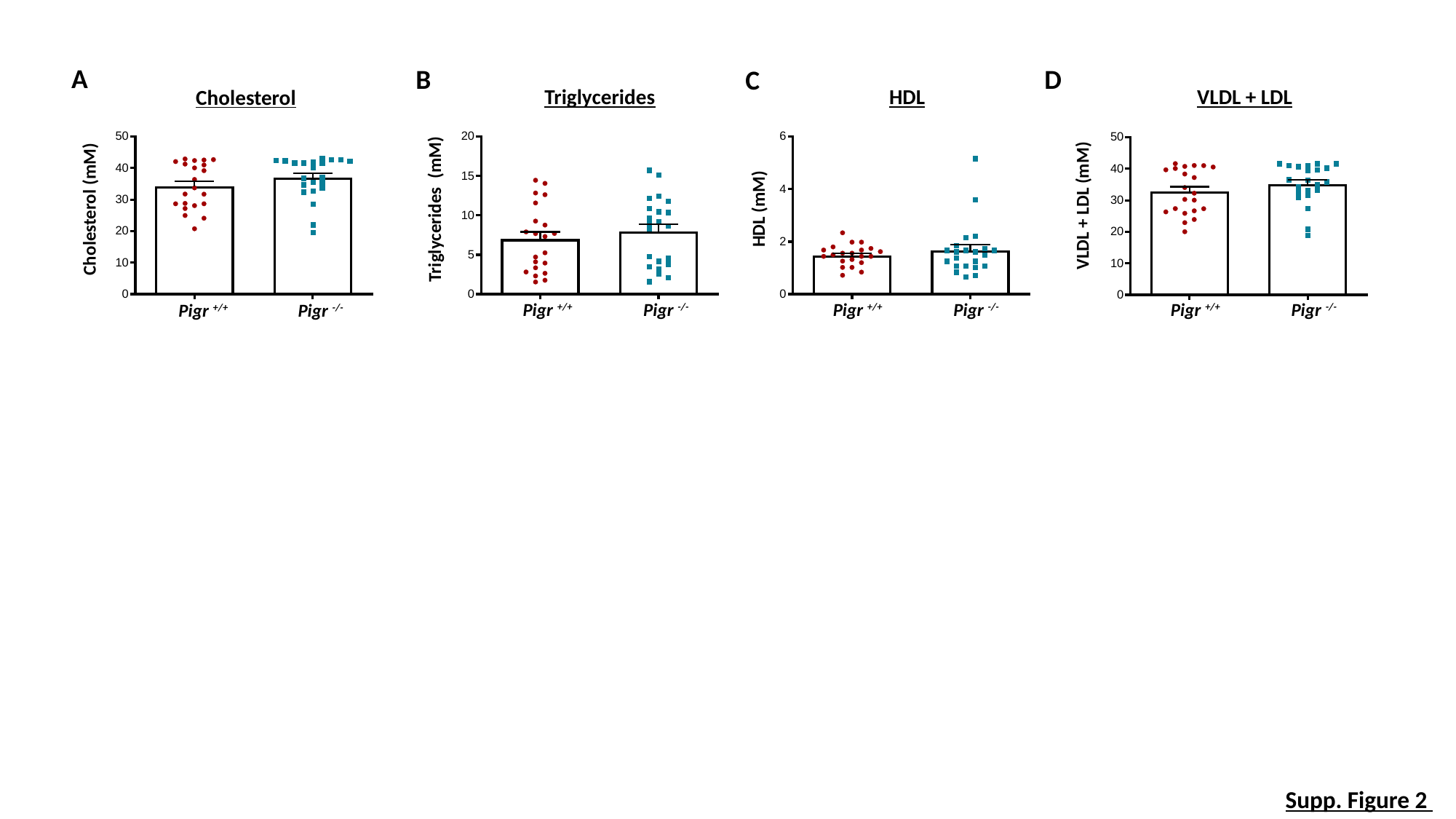

A
B
D
C
Triglycerides
HDL
VLDL + LDL
Cholesterol
VLDL + LDL (mM)
HDL (mM)
Triglycerides (mM)
Cholesterol (mM)
Pigr +/+
Pigr -/-
Pigr +/+
Pigr -/-
Pigr +/+
Pigr -/-
Pigr +/+
Pigr -/-
Supp. Figure 2

## Slide 3
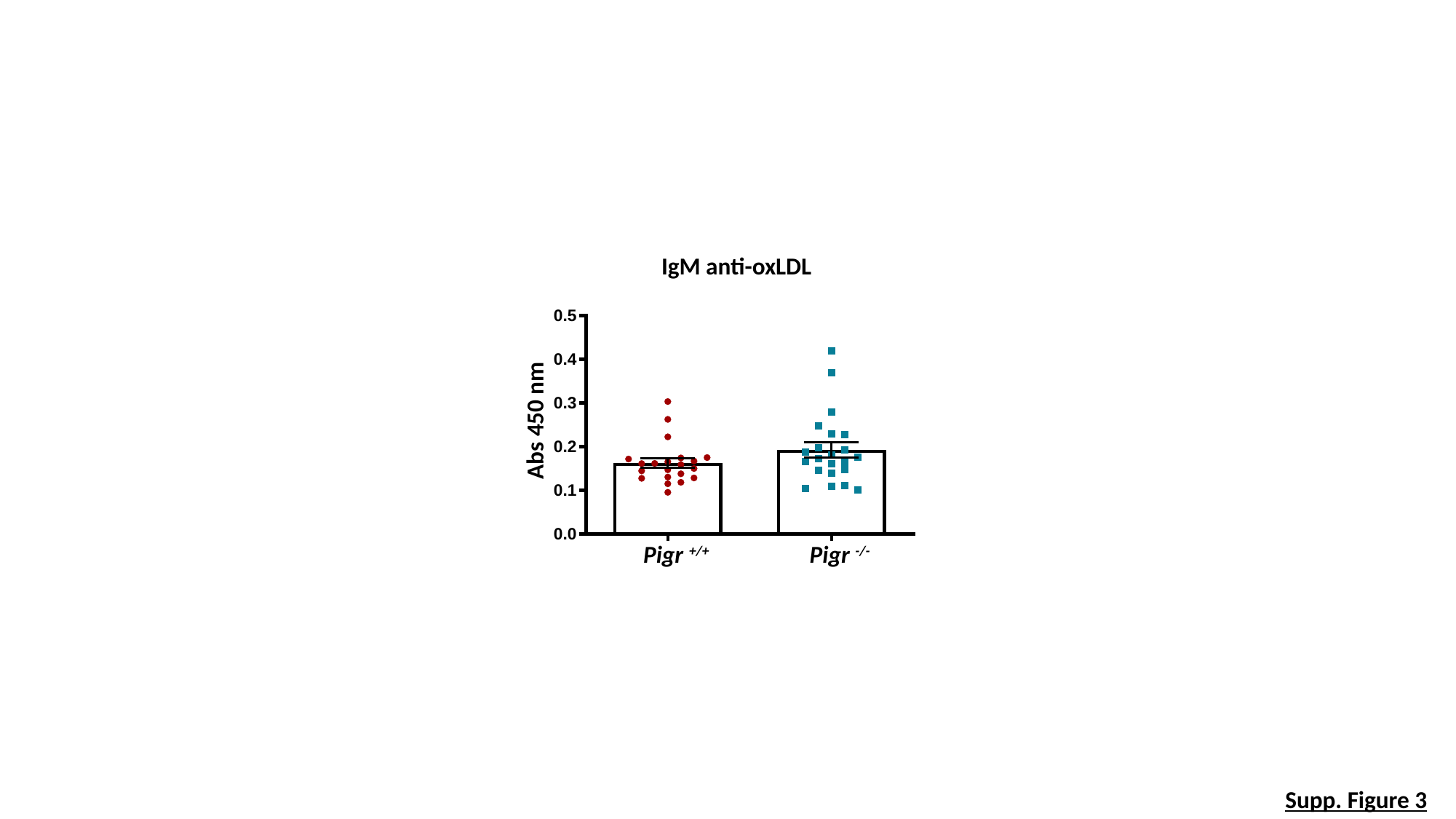

IgM anti-oxLDL
Abs 450 nm
Pigr +/+
Pigr -/-
Supp. Figure 3

## Slide 4
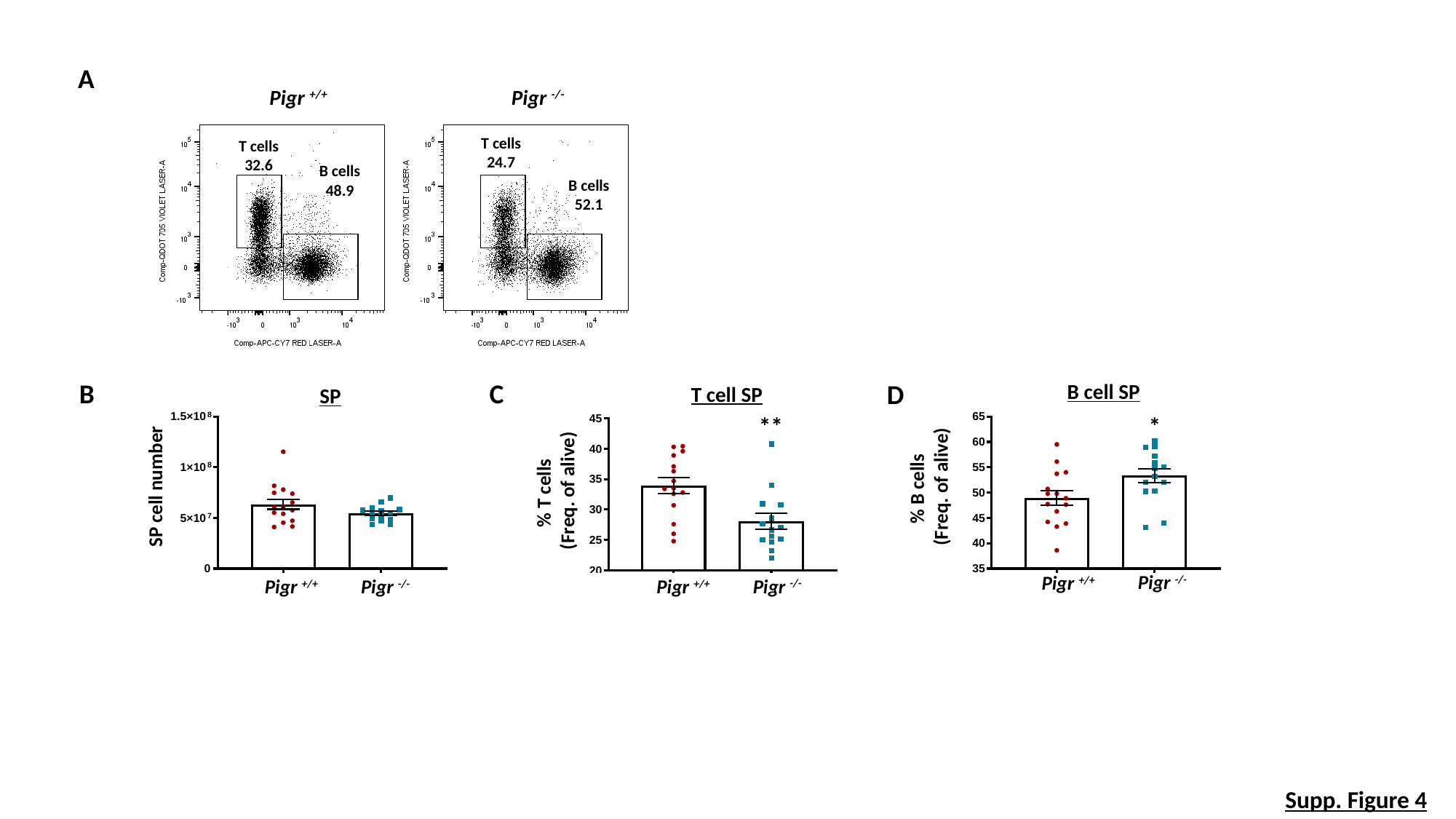

A
Pigr +/+
Pigr -/-
T cells
24.7
T cells
32.6
B cells
48.9
B cells
52.1
B
C
D
B cell SP
T cell SP
SP
**
*
% B cells
(Freq. of alive)
% T cells
(Freq. of alive)
SP cell number
Pigr -/-
Pigr +/+
Pigr -/-
Pigr -/-
Pigr +/+
Pigr +/+
Supp. Figure 4

## Slide 5
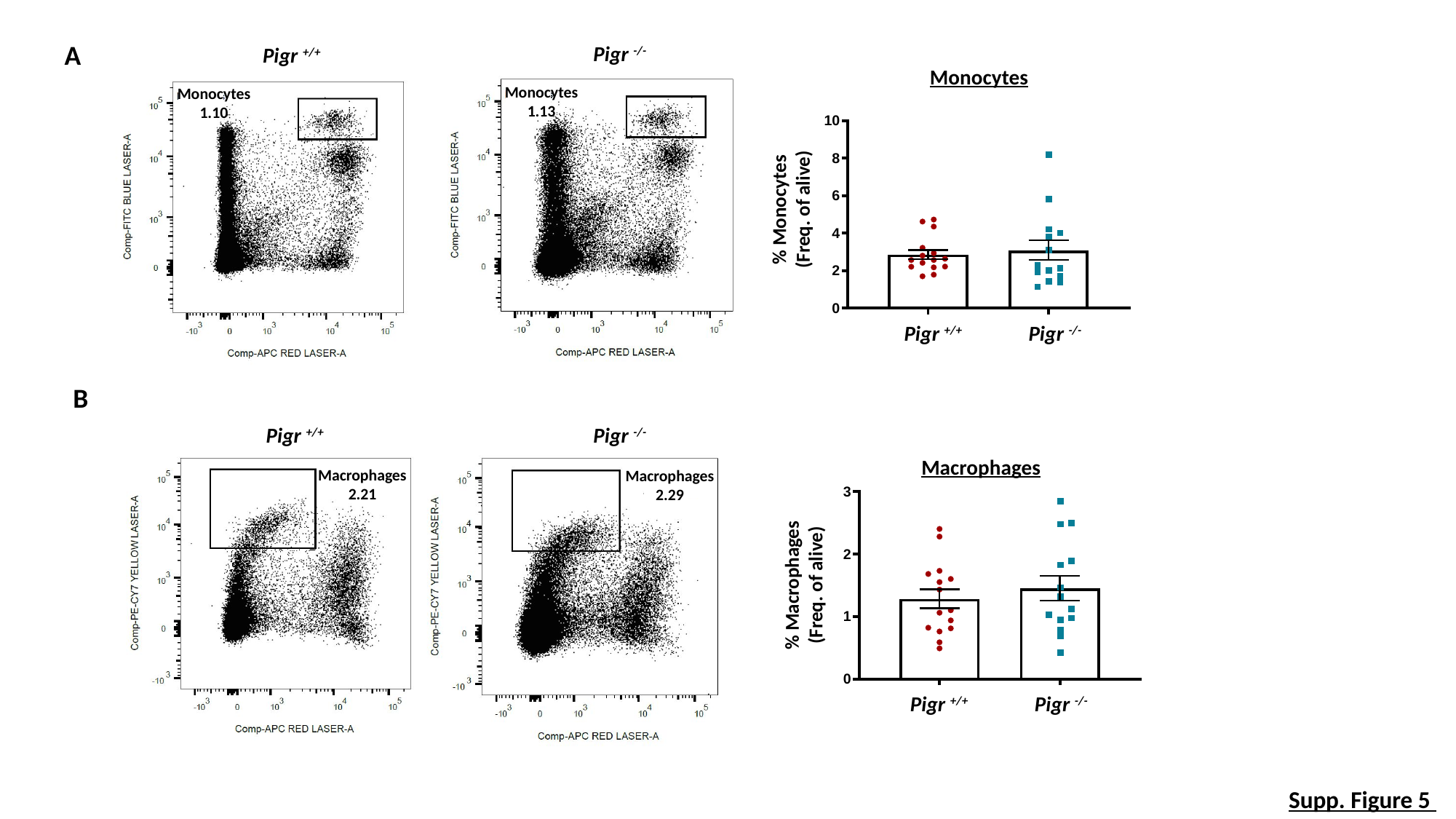

A
Pigr -/-
Pigr +/+
Monocytes
Monocytes
1.13
Monocytes
1.10
% Monocytes
(Freq. of alive)
Pigr +/+
Pigr -/-
B
Pigr -/-
Pigr +/+
Macrophages
Macrophages
2.21
Macrophages
2.29
% Macrophages
(Freq. of alive)
Pigr +/+
Pigr -/-
Supp. Figure 5

## Slide 6
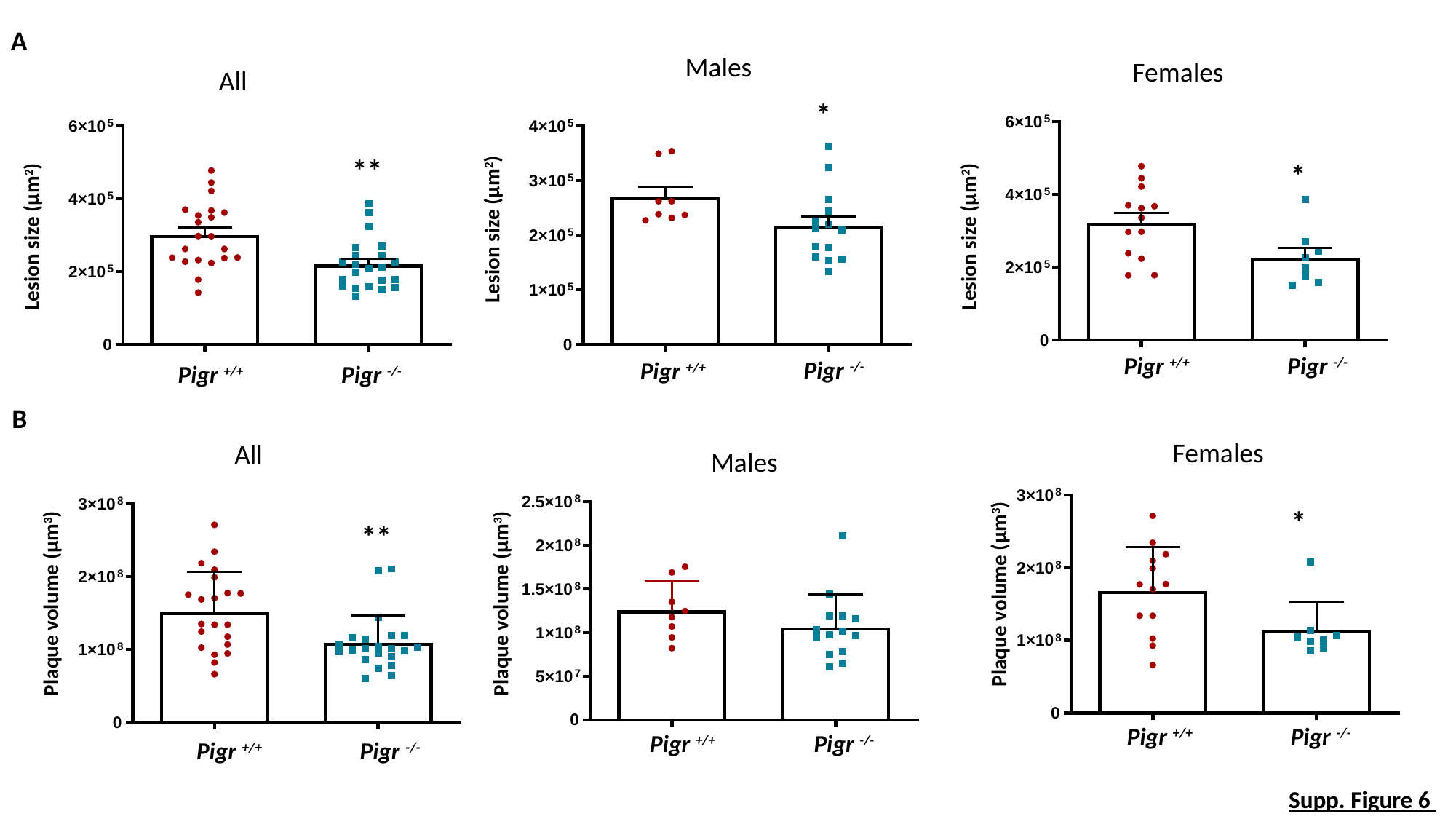

A
Males
Females
*
Lesion size (µm2)
Pigr -/-
Pigr +/+
All
*
**
Lesion size (µm2)
Lesion size (µm2)
Pigr -/-
Pigr +/+
Pigr -/-
Pigr +/+
B
Females
*
Plaque volume (µm3)
Pigr -/-
Pigr +/+
All
Males
Plaque volume (µm3)
Pigr -/-
Pigr +/+
**
Plaque volume (µm3)
Pigr -/-
Pigr +/+
Supp. Figure 6

## Slide 7
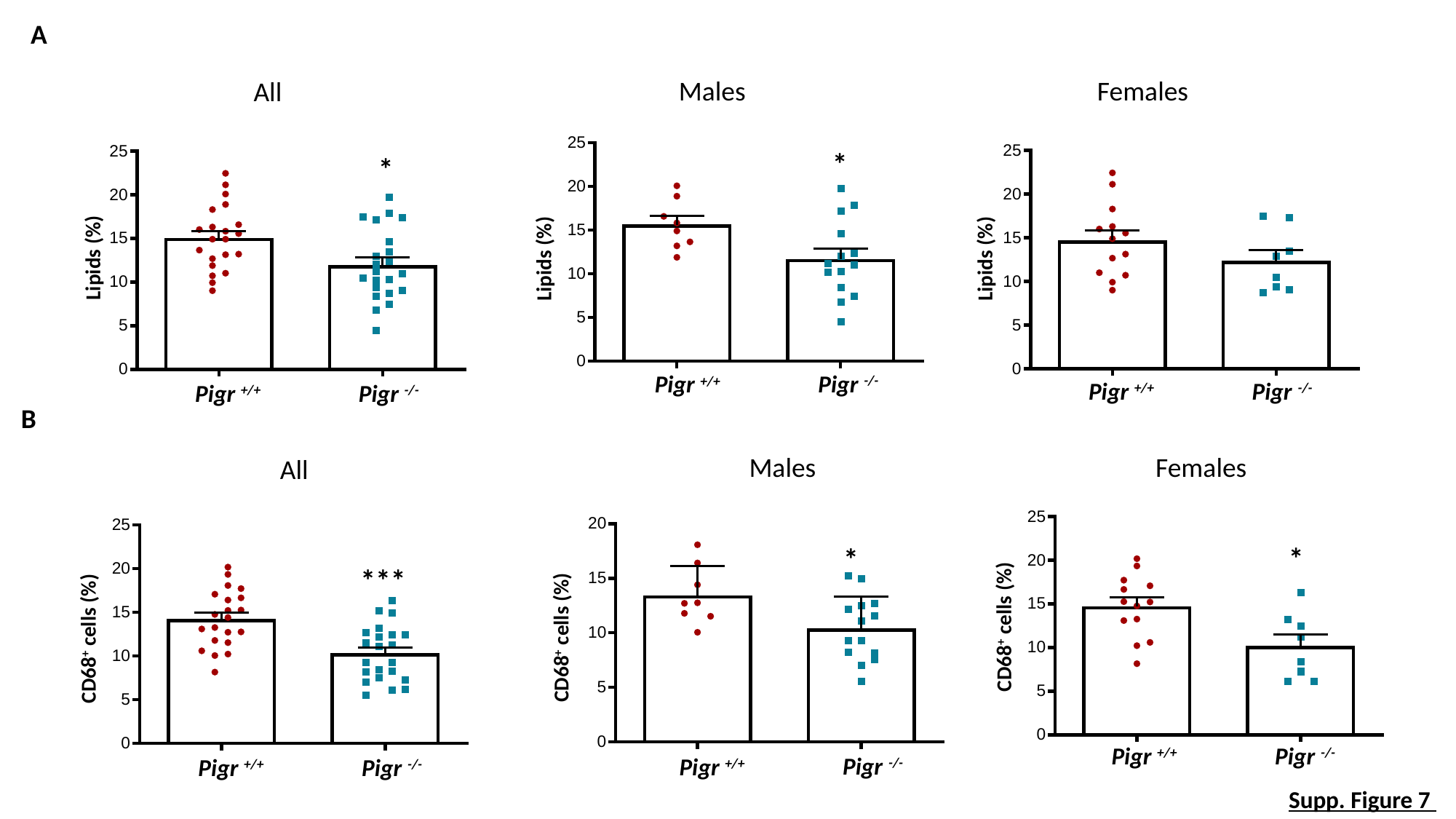

A
Males
Females
All
*
Lipids (%)
Pigr -/-
Pigr +/+
*
Lipids (%)
Lipids (%)
Pigr -/-
Pigr +/+
Pigr -/-
Pigr +/+
B
Males
Females
All
***
CD68+ cells (%)
Pigr -/-
Pigr +/+
*
*
CD68+ cells (%)
CD68+ cells (%)
Pigr -/-
Pigr +/+
Pigr -/-
Pigr +/+
Supp. Figure 7

## Slide 8
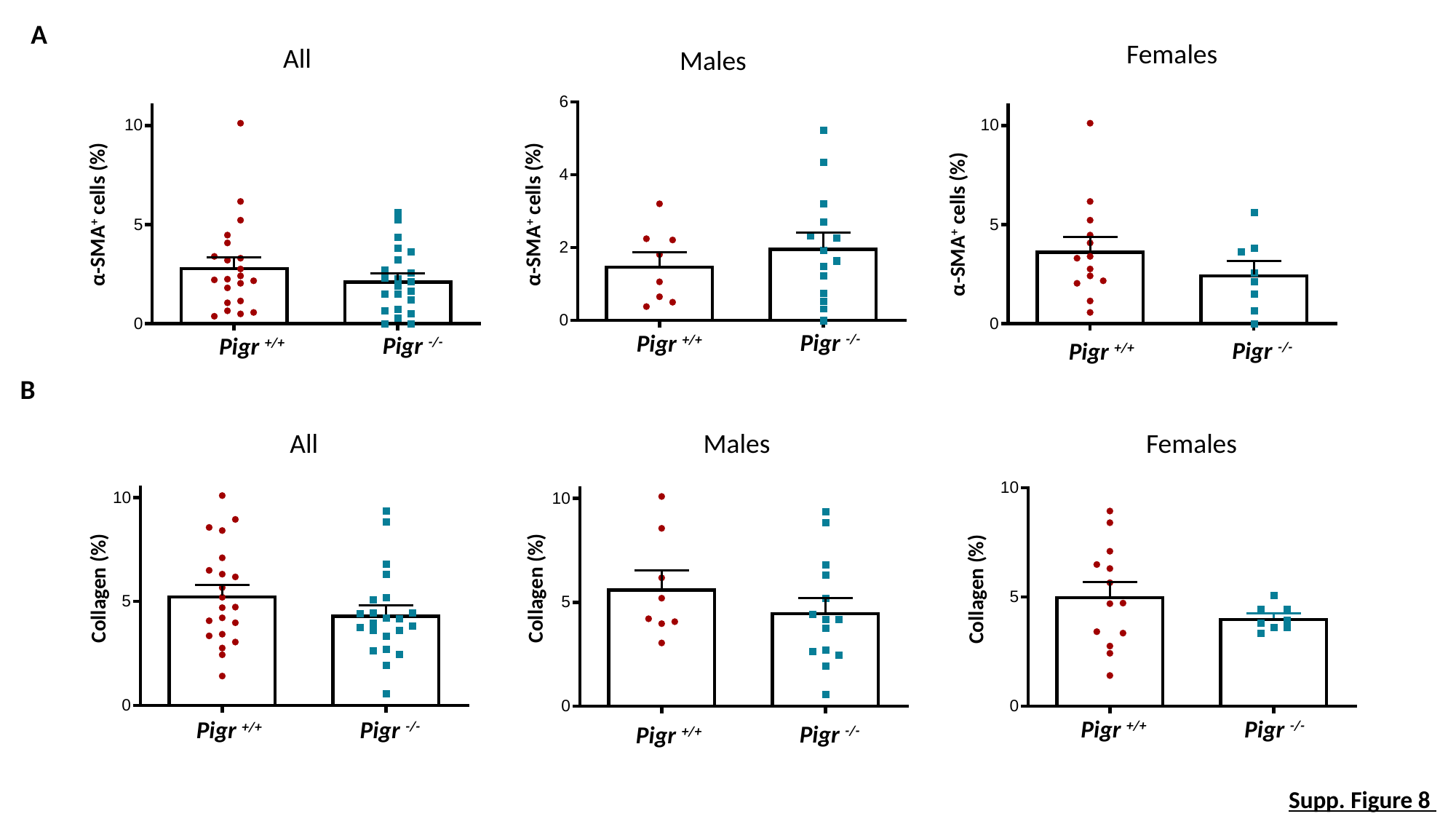

A
Females
α-SMA+ cells (%)
All
α-SMA+ cells (%)
Pigr -/-
Pigr +/+
Males
α-SMA+ cells (%)
Pigr -/-
Pigr +/+
Pigr -/-
Pigr +/+
B
All
Males
Females
Collagen (%)
Collagen (%)
Collagen (%)
Pigr -/-
Pigr +/+
Pigr -/-
Pigr +/+
Pigr -/-
Pigr +/+
Supp. Figure 8
